# Temporal Variations and Spatial Clusters of Dengue in Thailand: Longitudinal Study before and during the Coronavirus Disease (COVID-19) Pandemic

**DOI:** 10.3390/tropicalmed7080171

**Published:** 2022-08-08

**Authors:** Sayambhu Saita, Sasithan Maeakhian, Tassanee Silawan

**Affiliations:** 1 Faculty of Public Health, Thammasat University, Lampang 25190, Thailand; 2 Thammasat University Research Unit in One Health and Ecohealth, Thammasat University, Pathum Thani 12120, Thailand; 3 Division of Epidemiology, Department of Disease Control, Ministry of Public Health, Nonthaburi 11000, Thailand; 4 Department of Community Health, Faculty of Public Health, Mahidol University, Bangkok 10400, Thailand

**Keywords:** dengue, temporal variations, spatial clusters, coronavirus disease, COVID-19

## Abstract

The efforts towards effective control of the COVID-19 pandemic may affect the incidence of dengue. This study aimed to investigate temporal variations and spatial clusters of dengue in Thailand before and during the COVID-19 pandemic. Reported dengue cases before (2011–2019) and during (2020–2021) the COVID-19 pandemic were obtained from the national disease surveillance datasets. The temporal variations were analyzed using graphics, a seasonal trend decomposition procedure based on Loess, and Poisson regression. A seasonal ARIMA model was used to forecast dengue cases. Spatial clusters were investigated using the local indicators of spatial associations (LISA). The cyclic pattern showed that the greatest peak of dengue cases likely changed from every other year to every two or three years. In terms of seasonality, a notable peak was observed in June before the pandemic, which was delayed by one month (July) during the pandemic. The trend for 2011–2021 was relatively stable but dengue incidence decreased dramatically by 7.05% and 157.80% on average in 2020 and 2021, respectively. The forecasted cases in 2020 were slightly lower than the reported cases (2.63% difference), whereas the forecasted cases in 2021 were much higher than the actual cases (163.19% difference). The LISA map indicated 5 to 13 risk areas or hotspots of dengue before the COVID-19 pandemic compared to only 1 risk area during the pandemic. During the COVID-19 pandemic, dengue incidence sharply decreased and was lower than forecasted, and the spatial clusters were much lower than before the pandemic.

## 1. Introduction

Dengue is an infectious disease caused by the dengue virus (DENV), which belongs to a single-strand RNA arbovirus (family, Flaviviridae; genus, *Flavivirus*). Dengue consists of four serotypes, DENV-1, DENV-2, DENV-3, and DENV-4, and can cause disease in humans [1]. *Aedes aegypti* and *Aedes albopictus* mosquitoes are the primary vectors in the tropical zone [2]. The infection has an incubation period of 4–10 days after a person has been bitten by an *Aedes* mosquito, and 80–90% of infected people are asymptomatic. Of the 10–20% with clinical symptoms, 50% have mild clinical symptoms, 40% are dengue fever (DF) patients and 10% have severe clinical symptoms classified as dengue hemorrhagic fever (DHF), of which 5–30% are characterized as having dengue shock syndrome (DSS) [3].

The global incidence of dengue has increased dramatically in the past decade, and it has been estimated that 3.9 billion people are at risk of infection. The incidence of dengue increased over eightfold from 2000 to 2019, but has likely declined during the coronavirus disease 2019 (COVID-19) pandemic (2020–2021) [1]. Thailand has been reporting dengue outbreaks for a long time and has timescales of multiannual oscillations that vary in space and time [4]. The epidemic has spread throughout the country, and dengue cases have been reported in every province and region. The dengue fatality rate during the COVID-19 pandemic was approximately 0.1% [3], which is lower than that in previous years and is lower than the global rate. However, the morbidity rates were still high and do not seem to have decreased. The incidence rates of DF, DHF, and DSS in 2020 were 76.66, 31.71, and 0.83 per 100,000 population, and these cases were mainly found in the north, northeast, and south regions, respectively [5].

COVID-19 is a global threat that reportedly started in China at the end of 2019 and thereafter spread to several countries. The World Health Organization (WHO) declared COVID-19 a global pandemic on 11 March 2020 [6]. Various control and preventive measures were taken in each country, including city-wide lockdowns that had effects on the transmission of other infectious diseases, such as pneumonia, influenza, and dengue, leading to a decreased incidence of these during the period of the COVID-19 pandemic [7]. In addition, statistically significant and homogeneous reductions in the risk of dengue were observed at all levels during the lockdown in Sri Lanka [8]. In Thailand, during the first wave of the pandemic (1 January–14 December 2020), 4237 confirmed cases of COVID-19 were detected, with 60 deaths [9]. During the second wave, the number of confirmed cases increased 300 times compared to those observed during the first wave and, at present (8 July 2022), the number of confirmed cases has increased by about 1000-fold compared with the first wave [10]. The proportion of deaths was high in people who had an underlying disease and/or who were elderly [10]. Public health, social, and other measures from all departments at the central, local, and community levels were mobilized, integrated, and vigorously implemented to control and prevent the spread of the disease and to save patients’ lives [11,12]. The COVID-19 pandemic not only affected human behavior, but also affected human movement, transportation, and the length of time spent indoors during the day [12]. These are closely related to the occurrence of dengue, which is influenced by population movement, the residential environment, meteorological indices, and human–mosquito contact [13,14,15,16]. Moreover, some limitations during COVID-19, such as health-seeking behaviors for probable cases, misdiagnosis in patients with flu-like symptoms, and the inability to confirm the diagnosis using laboratory tests due to insufficient equipment and labor, resulted in underreporting [8,17].

In China, the dengue epidemic in Yunnan during the COVID-19 pandemic dramatically decreased compared to non-pandemic years, and the preventive measures against COVID-19 were efficient at preventing the transmission of dengue between cities and from urban to suburban areas [18]. Studies using time series modeling showed that the predicted dengue cases during the COVID-19 pandemic were higher than the reported cases in many countries in Latin America (i.e., Dominican Republic and Jamaica), Southeast Asia (i.e., Cambodia and Philippines) [16], and South Asia (i.e., Sri Lanka) [19]. On the contrary, the dengue cases in Peru increased in many endemic regions during the COVID-19 pandemic, with the highest incidence ratio (IRR) being 90.14 [15]. The observed cases in some countries (e.g., Brazil) were also higher than in model predictions [16].

There is no specific antiviral treatment for dengue. An anti-dengue vaccine has recently been introduced, but due to its inconsistent efficacy and safety issues, its use is restricted to specific target groups [20]. Therefore, dengue control still primarily relies on vector control, environmental management, population movements, and other social determinants. Thus, well-designed, reliable strategies to identify the temporal variations, risk areas or hotspots, and forecasted cases are needed, particularly during the COVID-19 pandemic. The current research aimed to investigate temporal variations and spatial clusters of dengue in Thailand before and during the COVID-19 pandemic. The findings provide an understanding of how the disease situation has changed over time and space both before and during the COVID-19 pandemic. In addition, the findings could orient future planning as well as the implementation of dengue prevention and control measures that are appropriate for the temporal variations and spatial clusters.

## 2. Materials and Methods

### 2.1. Study Design and Study Areas

**Figure 1:**
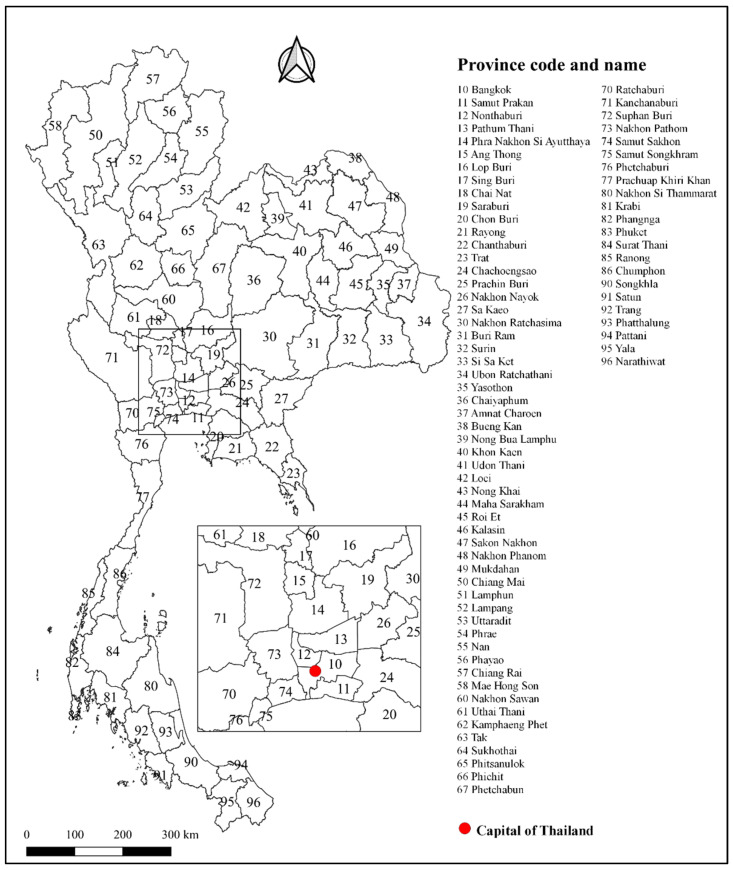
Map of the 77 provinces in Thailand, including the Bangkok Metropolitan Administration.

A longitudinal study was carried out using retrospective data collected at the province level. The study areas were seventy-seven provinces (including Bangkok, the capital city) in Thailand (Figure 1). Thailand, officially known as the Kingdom of Thailand, is located in Southeast Asia, bordered by Laos People’s Democratic Republic (PDR) and Cambodia to the east, the Gulf of Thailand and Malaysia in the south, the Andaman Sea and Myanmar in the west, and Laos PDR and Myanmar in the north. It has a land area of approximately 513,120 km^2^ and a total population of almost 70 million. There are six provinces in Bangkok and its vicinities, six provinces in the central region, eight provinces in the eastern region, six provinces in the western region, seventeen provinces in the northern region, twenty provinces in the northeastern region, and fourteen provinces in the southern region. The majority of the population is composed of Thai nationals (98.6%) and Buddhists (93.5%). The major economic sectors are agriculture, manufacturing, tourism, services, and natural resources. The transportation system, infrastructure, and communication systems are well-developed [21]. Thailand’s climate can be divided into three seasons: summer from mid-February to mid-May, the rainy season from mid-May to mid-October, and winter from mid-October to mid-February, except in the southern region, where only summer and the rainy seasons are present [22].

### 2.2. Dengue Data

New cases of dengue diagnosed according to the 1997 WHO guidelines [23] are reported by the local health facilities to the national disease surveillance system using the R506 form [5]. Dengue is classified into DF, DHF, and DSS. DF is classified as a patient who has 2–7 days of acute fever with at least two of the following: headache, retro-orbital pain, myalgia, arthralgia, rash, hemorrhagic manifestations (positive tourniquet test, bleeding spots, nosebleed), white blood cell count ≤ 5.0 × 10^9^/L with an increase in atypical lymphocytes, an increase in hematocrit of 5–10%, and platelet count ≤ 150 × 10^9^/L. DHF is classified as a patient with 2–7 days of acute and persistent high fever and the clinical features of hemorrhagic manifestations, thrombocytopenia, and laboratory evidence of plasma leakage. DSS is classified as a patient who meets all DHF criteria and shows evidence of circulatory failure and hypotension with tissue hypoperfusion [3,23]. WHO revised the guidelines in 2009 and classified dengue cases according to their severity into dengue (dengue without warning signs: DF or DHF, Grades I and II) and severe dengue (dengue with warning signs: abdominal tenderness, persistent vomiting, and clinical fluid accumulation) [24].

The datasets of monthly reported dengue cases and the midyear population in the 77 provinces before the COVID-19 pandemic from January 2011 to December 2019 and during the COVID-19 pandemic from January 2020 to December 2021 were obtained from the national disease surveillance system available on the website of the Division of Epidemiology, Ministry of Public Health [5].

### 2.3. Temporal Analysis

The temporal variations were analyzed using RStudio version 1.1.419 (RStudio Team, Boston, MA, USA) [25]. Line graphs were generated for visual interpretation of the cyclic variations, and box plots were drawn to see the seasonal variations more clearly. A seasonal trend decomposition procedure based on Loess (STL) was generated to analyze disease trends after adjusting for seasonal influences and to analyze seasonality after adjusting for trend influences. The adjusted data series of the trend was analyzed using a Poisson regression model to determine the change in dengue cases over years in which monthly cases were the outcome, population size was the offset term, and month was the predictor variable.

The non-stationarity of the data series was overcome by transforming and differencing methods. The seasonal autoregressive integrated moving average (seasonal ARIMA or SARIMA) models, (p,d,q,)(P,D,Q)^s^, using the Box–Jenkins approach were generated [26], where p and P refer to the order of the non-seasonal (or ordinary) and seasonal autoregressive (AR) models, d and D refer to the order of non-seasonal and seasonal differencing, q and Q refer to the order of the non-seasonal and seasonal moving average (MA) models, and s refers to the length of a season (12 months). The autocorrelation function (ACF) and the partial autocorrelation function (PACF) were the tools used to identify the order of the AR and MA in the tentative models.

To forecast dengue cases during the COVID-19 pandemic (January 2020 to December 2021) compared with the actual reported cases, tentative seasonal ARIMA models were identified and fitted to data series of monthly dengue cases from 2011 to 2018 (the training set) and the cases from January to December 2019 (the validating set) using the one-step-ahead prediction method. A model diagnostic test was performed in which the models with significant parameters (*p* < 0.05), those for which the residual of ACF was statistically equal to zero or white noise, those with *p*-values for the Ljung–Box statistics of >0.05, and those with a low Akaike information criterion (AIC) were considered to be adequate models. The forecasted cases from those adequate models were compared with the actual reported cases, and the mean absolute percentage error (MAPE) was calculated for the model accuracy, in which the model with the least MAPE was the model with the best fit. The best fitting model was then fitted to the data from 2011 to 2019 to forecast the dengue cases from January 2020 to December 2021.

Using the same approaches, the tentative seasonal ARIMA models were identified and fitted to the data series from 2011 to 2019 (the training set), and the cases from January 2020 to December 2021 (the validating set) were forecasted. The best fitting model was then fitted to the data from 2011 to 2021 to forecast the dengue cases from January to December 2022.

### 2.4. Spatial Analysis

Spatial analysis and mapping were performed using QGIS 3.20 (QGIS Development Team, Malaga, Spain) [27] and GeoDa 1.18.0 (Luc Anselin, IL, USA) [28]. The annual incidence rates per 100,000 population of dengue from 2011 to 2021 at the province level were calculated and mapped for visual interpretation, comparing between before (2011–2019) and during the COVID-19 pandemic (2020–2021). The spatial empirical Bayesian (SEB) approach was used to minimize the phenomenon of the modifiable areal unit problem (MAUP) [29]. SEB is one of the smoothing methods for disease rates; it is used for solving the problem of comparing rates in different population sizes related to the problem of variance instability and spurious outliers. SEB smoothing addresses variance instability by borrowing strength from other spatial units and the smoothing of disease rates for mapping in small areas enhances the visualization of spatial patterns. The SEB-smoothed rates per 100,000 population were also calculated and mapped for visual interpretation, comparing between before (2011–2019) and during the COVID-19 pandemic (2020–2021).

The spatial weight used in this analysis, which indicates whether regions are neighbors to each other, was the K-nearest neighbor method with four neighbors. The local clusters based on SEB-smoothed rates of dengue were investigated using the Moran local indicators of spatial association (Moran LISA or LISA) statistics [30]. LISA detects clusters of either similar or dissimilar disease frequency values around a given observation, which means that a LISA is an indicator of the extent to which the value of an observation is similar to or different from its neighboring observations. The LISA cluster map provides essential information on the significant locations by the type of spatial autocorrelation. The four types are high–high (red area: areas that have high rates and have neighbors that also have high rates), low–low (blue areas: areas that have low rates and have neighbors that also have low rates), high–low (pink areas: areas that have high rates and have neighbors that have low rates), and low–high (pale blue: areas that have low rates and have neighbors that have high rates). The significance is based on 99 random permutation procedures, with *p* < 0.05 considered to be significant.

## 3. Results

### 3.1. Temporal Variation

#### 3.1.1. Cyclic, Seasonal, and Trend

**Figure 2:**
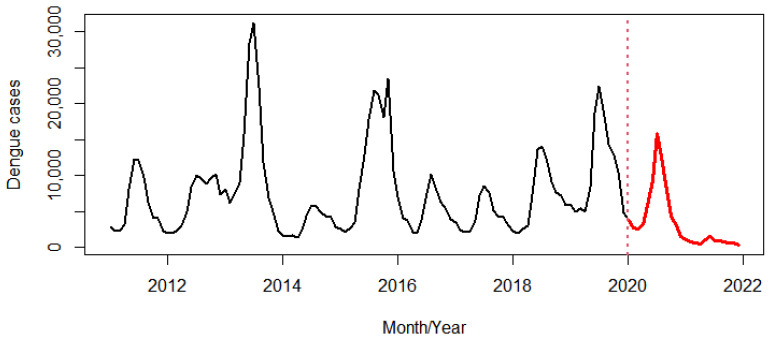
Dengue incidence before (black line) and during the COVID-19 pandemic (red line). The red dots represent the beginning of the COVID-19 pandemic in Thailand.

**Figure 3:**
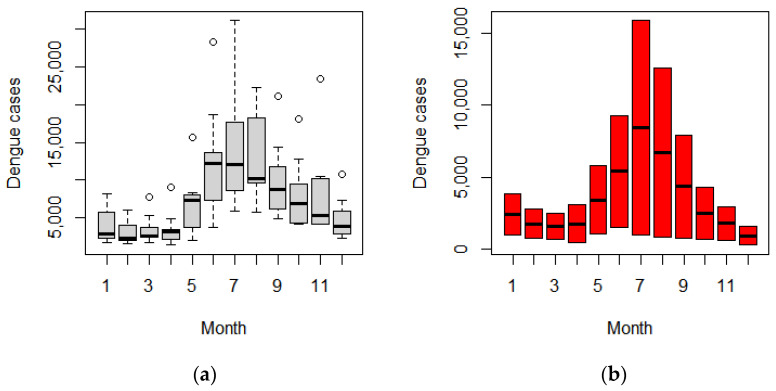
Seasonal pattern of dengue incidence. Seasonal boxplot before; the circles refer to outlier values (**a**) and during (**b**) the COVID-19 pandemic.

During the first period, a large peak in dengue cases was observed every other year (in 2013 and 2015). Thereafter, this changed to two consecutive low peaks from 2016 to 2017 and three consecutive high peaks from 2018 to 2020 (Figure 2). Before the COVID-19 pandemic, a seasonal pattern of dengue incidence showed a large peak from July to August and a smaller peak in November. By contrast, there was only one peak in July during the pandemic (Figure 3).

**Figure 4:**
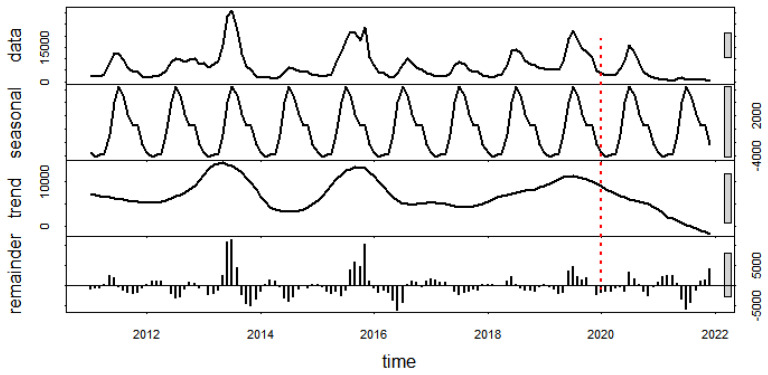
The STL of dengue incidence in Thailand from 2011 to 2021; raw data (top), seasonal component (second from top), trend component (third from top), and residual or remainder (bottom). The red dots represent the beginning of the COVID-19 pandemic in Thailand.

After adjusting for seasonal variation and removing background noise from the data series, the regression coefficients indicated that the trend for dengue incidence from 2011 to 2021 was relatively stable, with a 0.27% reduction for every one additional month of the study period (IRR 0.9988, 95% CI 0.9973–1.0004). If we consider the period during the COVID-19 pandemic only, the dengue incidence decreased dramatically, with a 7.05% and 157.80% reduction on average from the previous month in 2020 and 2021, respectively (Figure 4).

#### 3.1.2. Forecasting Dengue Incidence

**Figure 5:**
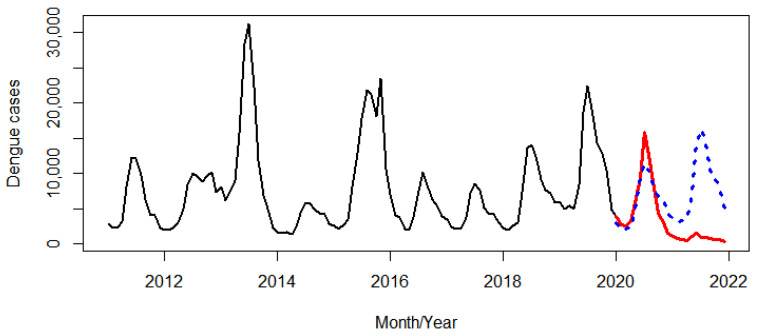
Forecasted dengue cases in 2020–2021 (blue dots), and dengue cases before (black line) and during the COVID-19 pandemic (red line).

The seasonal ARIMA (2, 0, 0)(2, 1, 0)^12^ model with a MAPE of 1.58% was fitted to monthly dengue cases from 2011 to 2019 (before the COVID-19 pandemic) to predict dengue cases from January 2020 to December 2021 (during the COVID-19 pandemic). The forecasted dengue cases in the year 2020 and 2021 were 70,638 and 98,239 cases, respectively, compared with the actual reported cases: 72,519 (2.63% more than the forecast) and 9956 (163.19% less than the forecast) in the corresponding years (Figure 5).

**Figure 6:**
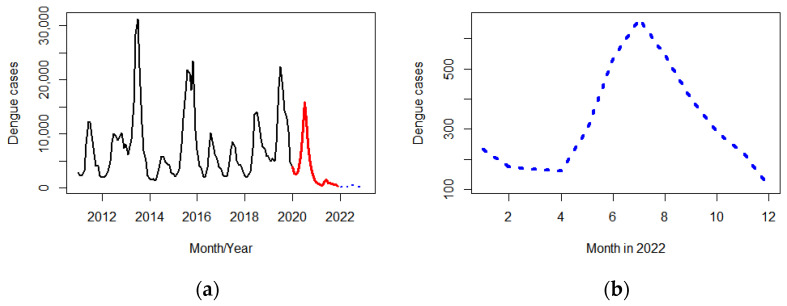
Forecasted dengue cases in 2022 (blue dots), and dengue cases before (black line) and during the COVID-19 pandemic (red line) (**a**). An enlarged view of forecasted dengue cases in 2022 (**b**).

The seasonal ARIMA (1, 1, 0)(2, 1, 0)^12^ model with a MAPE of 13.96% was fitted to the whole data series from 2011 to 2021 and the dengue cases in 2022 were forecasted: 3810 cases with a peak in July (664 cases) (Figure 6).

### 3.2. Spatial Clusters

**Figure 7:**
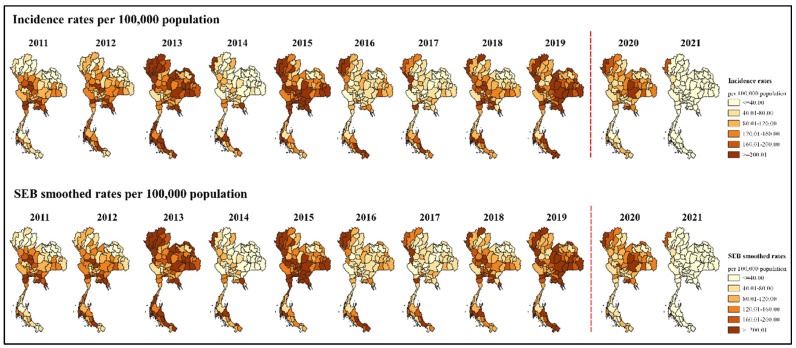
Map of the incidence rates (**top**) and the SEB-smoothed rates (**bottom**) of dengue before and during the COVID-19 pandemic. The red dots represent the beginning of the COVID-19 pandemic in Thailand.

Before the COVID-19 pandemic (2011–2019), the annual incidence rates of dengue were four times higher than the national target (40 cases per 100,000 population; a 10% decrease from the previous target of 50 cases per 100,000 population) in about half of all provinces during the high peak years (2013, 2015, 2019) and were 7.8–32.5% in the low peak years. In contrast to the previous years, the high incidence rates (four times higher than the national target) appeared in 1.3% of the provinces in 2020 and in none in 2021. There were 10 provinces in 2020 (namely, Mae Hong Son, Chiang Mai, Nakhon Ratchasima, Chaiyaphum, Surin, Khon Kean, Bueng Kan, Chai Nat, Rayong, and Ang Thong) and only 1 province in 2021 (Mae Hong Son) where the incidence rate was four times higher than the national target (Figure 7).

After minimization of the variance instability due to the phenomenon of the MAUP using the SEB smoothing method and mapping of the annual SEB-smoothed rates in the same scale of the incidence rates, a difference between the two maps during the period before the COVID-19 pandemic was observed in the northeastern region: one province in 2015 (Yasothon) and one province in 2016 (Surin). During the pandemic, there were two provinces in the southern region (Prachuap Khiri Khan and Trang) where the SEB-smoothed rates differed from the incidence rates in 2020 (Figure 7).

**Figure 8:**
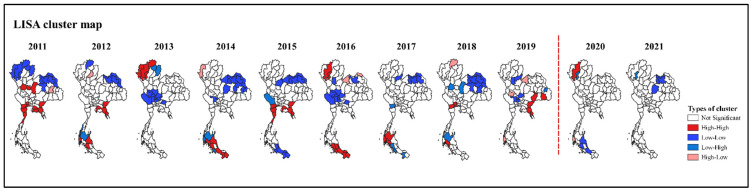
Map of the local indicators of spatial association (LISA) of the SEB-smoothed rates of dengue before and during the COVID-19 pandemic: high–high (red); low–low (blue); low–high (pale blue); high–low (pink). The red dots represent the beginning of the COVID-19 pandemic in Thailand.

Before the pandemic, the risk areas or hotspots (high–high clusters) of dengue from the LISA maps ranged from 5 to 13 provinces, while the lower risk areas (high–low clusters and low–high clusters) ranged from one to six provinces. The dengue hotspots were mainly located in the southern region in 5 years (2012, 2014, 2016, 2017, and 2018), followed by the eastern region in four years (2011, 2012, 2015, and 2019). During the pandemic, the LISA maps indicated significant local clusters in 2020, where one risk area (high–high clusters) and one lower-risk area (low–high clusters) occurred in the northern region (Chiang Mai and Lamphun Province, respectively), and one lower-risk area (high–low clusters) was observed in the southern region (Phang Nga Province). There was only one lower-risk area (low–high clusters) in 2021 in Lamphun Province (Figure 8).

## 4. Discussion

The seasonal peak of dengue before the COVID-19 pandemic was observed in June, with a small peak in November, but during the pandemic, a single peak occurred a month later (i.e., in July) compared with the pre-COVID-19 period. This seasonal delay can be explained by the climate, as it has been reported in Thailand [31] that a 1- to 3-week lag in precipitation was associated with a corresponding delay in the occurrence of the peak incidence of dengue [31]. The amount of precipitation can substantially affect mosquitos’ habitat by increasing water storage for laying eggs and may also correspond to the life cycle of mosquitos, as it takes up to 14 days for a female mosquito to become fully mature. On the other hand, the seasonal delay during the COVID-19 pandemic may not have been caused by the natural history of the disease but by delays in the diagnosis, laboratory-confirmed diagnosis, and health-seeking behavior of the probable cases. The small peak occurrence in November was probably due to rainfall in the short period before winter. This probably led to the creation of more *Aedes* spp. mosquito breeding sites and resulted in another peak of cases in November. In addition, having a long holiday in October encourages more people to travel or return to their hometowns, which can increase the number of infections [32,33,34].

The cyclic pattern of dengue in Thailand was usually observed every other year [35], meaning that the high peaks were expected and actually observed in 2013, 2015, and 2019, but not in 2017 and 2021. After 2015, the cyclic patterns seemed to appear every two or three years. This observation is consistent with the cyclical dengue outbreaks in Singapore [14] and Nepal [36]. The change in the dengue cycles is likely due to the shorter rainy season or rainfall days and the long winter, especially in 2017, due to La Niña [37] making the environment unsuitable for the mosquitoes and the spread of the disease. Moreover, extensive efforts to control and prevent the spread of the COVID-19 pandemic and to save patients’ lives [11,12] affected human behavior, travel, transportation, and length of stay at home [12], as well as some limitations, such as lack of access to health services and misdiagnosis [8,17], which are possible additional reasons that explain the low incidence of dengue in 2021. The trend of dengue derived from the STL, which decomposed the data for both periods (i.e., the entire study period from 2011 to 2021 and the period before the COVID-19 pandemic from 2011 to 2019), was stable. However, dengue cases during COVID-19 dramatically decreased, which was in agreement with the situation reported in Yunnan Province, China, which found that there was a substantial reduction of 96.20% relative to historical cases from the year before the pandemic [38].

The forecasted cases in 2020 were slightly lower than the reported cases, but in 2021, the reported cases were much lower than the forecasted cases. This may be because in 2020, the first year of the COVID-19 pandemic, COVID-19 had not spread widely in the country. The entire health system was still able to carry out planned actions in terms of health promotion, disease prevention, treatment, and rehabilitation, including disease reporting via the surveillance system. Apart from the reasons discussed earlier, the sharp decline in dengue incidence in 2021 might be due to the disruption-induced administrative delays in reporting [39]. In addition, the high peak dengue incidence in 2019 may be because 2019 was an unprecedented year for dengue globally, which led to high immunity against dengue infection [16,40] and resulted in fewer dengue cases being reported in 2020–2021. This is similar to the data on post-dengue outbreak years in Brazil [41]. Our findings are in agreement with the significant decrease in dengue cases in Southeast Asia and Latin America [16]. COVID-19 lockdowns and movement restrictions also led to decreased dengue transmission in Sri Lanka [19] and Brazil [42]. During the COVID-19 pandemic in Thailand, restrictions on movement within the country were introduced from 26 March 2020 to control the spread of COVID-19, especially closing schools, universities, and offices. During the lockdown, the dengue prevention and control program was interrupted. Public health staff and village health volunteers were heavily involved with COVID-19 mitigation activities, resulting in reduced activities related to dengue fever control as compared with the situation before the COVID-19 pandemic [43]. Moreover, people were staying or working at home and had more time to eliminate the breeding habitats of the *Aedes* mosquitoes both in the house and around the house. This resulted in a decrease in dengue incidence and may have affected the natural dengue cycle in 2021.

The forecasted dengue cases in 2022 from the seasonal ARIMA model showed the lowest number of cases compared with the previous decade and may differ from the natural history of dengue dynamics due to the COVID-19 pandemic, as described above [16]. The low number of forecasted cases may have been caused by the input of fewer cases during the COVID-19 pandemic from 2020 to 2021 into the forecasting model. Therefore, the results of the forecast might be underestimated, and evidence is needed to support the analysis to clarify whether this is because the number of cases is low or because of other contributing reasons regarding the limitations during COVID-19 such as the health-seeking behaviors of the probable cases, misdiagnosis of patients with flu-like symptoms, the inability to confirm the diagnosis using laboratory tests due to insufficient equipment and labor, and underreporting [8,17,39,44]. In addition, the Meteorological Department of the Ministry of Digital Economy and Society Thailand reported that Thailand’s average annual rainfall in 2018 was 1650.3 mm and dropped to 1343.4 mm in 2019. During the COVID-19 pandemic, rainfall increased slightly to 1527.3 mm and 1759.3 mm in 2020 and 2021, respectively [45,46,47,48]. These data support the possibility that the decline in dengue incidence was not due to reductions in the mosquito vectors. Thus, if effective prevention and control of the disease are not carried out, then a large epidemic of dengue may occur and may have a strong impact on people’s lives and the public health system.

Regarding the spatial analysis, there was a slight difference between the incidence rates and SEB-smoothed rates for each province and its four neighbors, according to the spatial weight used to minimize the variance instability in the analysis, which did not have much difference in population size. Therefore, both the incidence rates and SEB-smoothed rates can be used for prioritizing and allocating resources to each province.

In 2020, 10 provinces had dengue incidence rates more than four times higher than the national target. However, in 2021, the incidence in nine of these provinces was much lower than in the previous year, except for Mae Hong Son, where the morbidity rate was only three times lower than the previous year and the incidence rate was still more than four times higher than the national target. The reason for the sharp decline in the incidence of dengue in the nine provinces is probably due to 2021 being a year of low outbreak according to the natural disease cycle, as well as the impact of the COVID-19 pandemic, as discussed above. The incidence rate in Mae Hong Son Province did not decrease as much as other provinces and it still had a high rate. This is likely due to the majority of the population living in rural or remote areas, working in agriculture, and with sheltered sanitation which facilitates mosquito breeding sites [49], and because some houses lack protective equipment such as mosquito nets and repellents. Additionally, some populations are housed in valleys where humidity and the climate are suitable for mosquito breeding, consistent with previous studies that identified a relationship between dengue incidence and climate variables in Mae Hong Son Province [31]. In addition, Mae Hong Son’s precipitation data are consistent with the data of the entire country, declining in 2019 and increasing in 2021. However, in 2021, it was reported that Mae Hong Son had the highest temperature, compared with other provinces in the same region [50]. Moreover, the humidity comfort levels from May to November were at an oppressive and miserable level [51].

The southern and eastern regions were likely to be re-clustered areas during the period before the pandemic. However, a high-risk area during the pandemic was observed only in Chiang Mai in the northern region, which might be related to the high number of travelers. Chiang Mai is a famous province for both Thai and foreign travelers. Many people tend to visit during the festival, especially the Songkran festival (April) and winter season (November to December). In addition, relatively dense housing and 67% of people with knowledge of dengue have been reported in Chiang Mai [52]. The high–high cluster (areas that have high rates and neighbors that also have high rates) could be a dengue reservoir for spreading to the neighboring provinces. Moreover, there was a province with low incidence surrounded by a province with high incidence (low–high clusters) identified in Lamphun Province for two consecutive years. This may be because Lamphun is a small province located near Chiang Mai and is the gateway to Chiang Mai. Lamphun has cheaper accommodation and famous cultural attractions, which means that some tourists prefer to stay overnight in Lamphun before their trip to Chiang Mai or to stop by Lamphun after their Chiang Mai trip. This low–high outlier indicated areas at risk of infection spreading from the neighboring provinces if there is no effective control or good surveillance. Phang Nga Province in the southern region was a province with high incidence surrounded by provinces with low incidence (high–low clusters), which might be related to dengue being imported by travelers [53] due to the presence of many attractive places and the high number of travelers visiting those places in the early stages of the COVID-19 pandemic. Dengue from Phang Nga can spread to neighboring provinces if disease control in Phang Nga is inadequate or if prevention and surveillance measures in the neighboring provinces are ineffective.

Our results suggest that if the cyclic pattern changes to every 3 years, a large epidemic will occur in 2023. If the occurrence of dengue in 2017 is an unusual event, the cyclic pattern may be the same as previous periods (every other year), and a large epidemic should have been observed in 2021 and can be expected in 2023. Therefore, effective planning and preparedness should be well organized to control the disease in 2023, since the delayed seasonal peak during the COVID-19 pandemic may be due to factors other than the natural history of the disease. If the number of patients begins to increase in May, control measures should be strengthened a month prior to the seasonal peak in April, in accordance with the vector dynamics and incubation period of the disease. Control measures must also be strengthened a month before November, in which another small seasonal peak was observed. The forecasted cases in 2022 were also as low as in 2021, as the low case numbers in 2021 were fitted to the forecasting model. However, the reported cases could be higher than the forecasted cases because the control measures for COVID-19 in 2022 are less serious and lockdowns have stopped. This has enabled people to travel more for tourism, to return home, and to visit relatives and friends. Preparedness for increased cases of dengue in 2022 should also be launched in April to July.

For future studies, an interrupted time series analysis, as well as forecasting, should be explored by excluding the unusual low-peak years (such as 2017 and 2021) from the dataset to see the real natural history of the disease. An analysis of the synchrony of all pairs of the provinces throughout the country should be investigated to identify the external and internal factors contributing to dengue occurrence. The differences in the factors for the different cluster types should also be investigated for effective intervention. Longitudinal studies after the COVID-19 pandemic should be set up to closely observe the occurrence and natural history of dengue.

## 5. Conclusions

The cyclic pattern of dengue cases has likely changed from every other year to every two or three years. During the COVID-19 pandemic, the seasonal peak was delayed by one month, and dengue incidence decreased dramatically and was much lower than forecasted. The spatial clusters were much lower during the COVID-19 pandemic compared to the situation before the pandemic.

## Data Availability

Not applicable.

## Abbreviations

ACF	Autocorrelation function
AIC	Akaike information criterion
AR	Autoregressive model
ARIMA	Autoregressive integrated moving average
CI	Confidence interval
COVID-19	Coronavirus disease 2019
DENV	Dengue virus
DF	Dengue fever
DHF	Dengue hemorrhagic fever
DSS	Dengue shock syndrome
IRR	Incidence rate ratio
LISA	Local indicators of spatial association
MA	Moving average model
MAPE	Mean absolute percentage error
MAUP	Modifiable areal unit problem
PACF	Partial autocorrelation function
SEB	Spatial empirical Bayesian
STL	Seasonal decomposition of time series by Loess
WHO	World Health Organization
